# (22*E*,24*R*)-3β,5α,9α-Trihy­droxy­ergosta-7,22-dien-6-one monohydrate

**DOI:** 10.1107/S160053681101347X

**Published:** 2011-04-16

**Authors:** Zhi-Hong Xu, Xiao-Ping Peng, Yi Wang, Wei-Ming Zhu

**Affiliations:** a Key Laboratory of Marine Drugs of the Ministry of Education of China, School of Medicine and Pharmacy, Ocean University of China, 266003 Qingdao, People’s Republic of China

## Abstract

The title ergosterol compound, C_28_H_44_O_4_·H_2_O, is composed of four fused rings (three six-membered and one five-membered) and a side chain. It is a derivative of ergosterol and was isolated from a marine-derived halotolerant fungus, *Cladosporium cladosporioides* PXP-49. In the crystal, mol­ecules are assembled by classical O—H⋯O hydrogen bonds, forming a two-dimensional network, with base vectors [100] and [010]. The absolute configuration was assigned from the measured optical rotation and reference to the literature. An intra­molecular O—H⋯O hydrogen bond occurs.

## Related literature

For general background to the cytotoxic activity of similar compounds, see: Valisolalao *et al.* (1983 ▶); Kawagishi *et al.* (1988 ▶); Takaishi *et al.* (1991 ▶); Ishizuka *et al.* (1997 ▶); Yaoita *et al.* (1998 ▶); Sun *et al.* (2006 ▶); Tang *et al.* (2007 ▶); Cui *et al.* (2010 ▶). For details of ring conformations and puckering parameters, see: Cremer & Pople (1975 ▶); Baginski *et al.* (1989 ▶); Gonzalez *et al.* (2002 ▶).
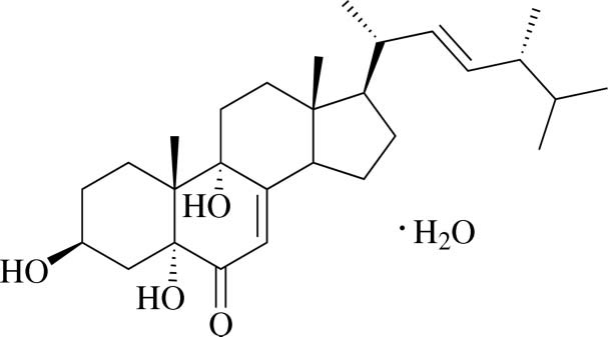


## Experimental

### 

#### Crystal data

C_28_H_44_O_4_·H_2_O
*M* *_r_* = 462.65
Monoclinic, 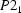

*a* = 6.7605 (8) Å
*b* = 7.2626 (11) Å
*c* = 28.461 (2) Å
β = 96.083 (1)°
*V* = 1389.5 (3) Å^3^
*Z* = 2
Mo *K*α radiation
μ = 0.07 mm^−1^
*T* = 298 K
0.40 × 0.35 × 0.17 mm

#### Data collection

Bruker SMART CCD area-detector diffractometer
Absorption correction: multi-scan (*SADABS*; Sheldrick, 1996 ▶) *T*_min_ = 0.971, *T*_max_ = 0.988
7040 measured reflections
2641 independent reflections
1557 reflections with *I* > 2σ(*I*)
*R*_int_ = 0.057

#### Refinement

*R*[*F* ^2^ > 2σ(*F* ^2^)] = 0.051
*wR*(*F* ^2^) = 0.108
*S* = 1.02
2641 reflections
304 parameters
1 restraint
H-atom parameters constrained
Δρ_max_ = 0.14 e Å^−3^
Δρ_min_ = −0.15 e Å^−3^

### 

Data collection: *SMART* (Bruker, 2007 ▶); cell refinement: *SAINT* (Bruker, 2007 ▶); data reduction: *SAINT*; program(s) used to solve structure: *SHELXS97* (Sheldrick, 2008 ▶); program(s) used to refine structure: *SHELXL97* (Sheldrick, 2008 ▶); molecular graphics: *XP* (Siemens, 1994 ▶); software used to prepare material for publication: *WinGX* (Farrugia, 1999 ▶).

## Supplementary Material

Crystal structure: contains datablocks global, I. DOI: 10.1107/S160053681101347X/su2267sup1.cif

Structure factors: contains datablocks I. DOI: 10.1107/S160053681101347X/su2267Isup2.hkl

Additional supplementary materials:  crystallographic information; 3D view; checkCIF report

## Figures and Tables

**Table 1:** Hydrogen-bond geometry (Å, °)

*D*—H⋯*A*	*D*—H	H⋯*A*	*D*⋯*A*	*D*—H⋯*A*
O1—H1⋯O5^i^	0.82	1.93	2.749 (4)	180
O2—H2⋯O5	0.82	1.90	2.716 (4)	177
O4—H4⋯O2	0.82	1.97	2.642 (4)	139
O5—H5*C*⋯O1^ii^	0.85	1.81	2.642 (4)	165
O5—H5*D*⋯O3^iii^	0.85	1.93	2.761 (4)	165

Symmetry codes: (i) 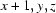; (ii) 
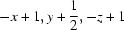
; (iii) 
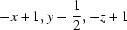
.

**Fig. 1.**
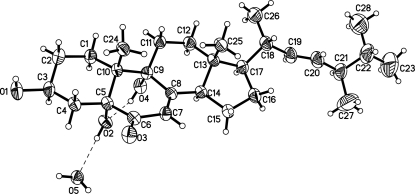
The molecular structure of the title molecule, with the atom-numbering scheme. Displacement ellipsoids are drawn at the 30% probability level. Dashed lines indicate the O-H···O hydrogen bonds (see Table 1 for details).

**Fig. 2.**
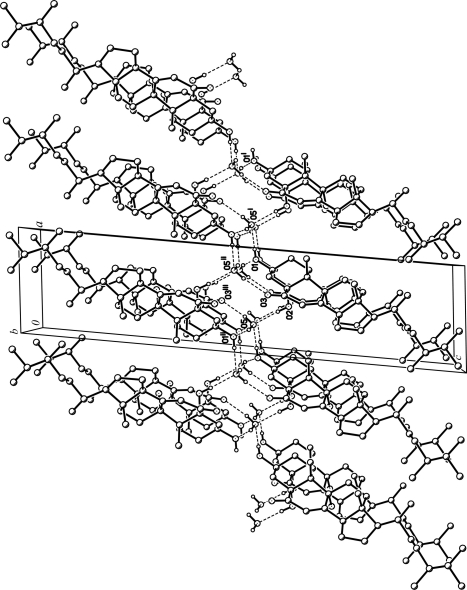
A view along the b-axis of the two-dimensional hydrogen-bonded network of the title compound, formed by O—H···O hydrogen bonds [dashed lines; see Table 1 for details; H-atoms not involved in hydrogen bonding have been omitted for clarity; Symmetry codes:(i) 1 + x, y, z; (ii) 1 - x, 1/2 + y, 1 - z; (iii) 1 - x, -1/2 + y, 1 - z].

## supplementary crystallographic information

### Comment

The title ergosterol compound was first isolated from the fungus *Polyporus versicolor* as a cytotoxic principle to hepatoma cells (Valisolalao *et al.*, 1983). This compound, and other related compounds from different genera of mushrooms, for eg. *Bugula neritina L*., and endophytic fungi derived from  marine red alga, are of interest due to their cytotoxic activity (Kawagishi *et al.*, 1988; Ishizuka *et al.*, 1997; Takaishi *et al.*, 1991; Yaoita *et al.*, 1998; Sun *et al.*, 2006; Tang *et al.*, 2007; Cui *et al.*, 2010). We isolated the title compound as part of our ongoing studies on characterizing bioactive metabolites from marine-derived halotolerant fungi, and we report herein on its crystal structure.

As illustrated in Fig. 1 the title compound is composed of four fused rings, three six-membered and one five-membered, and a side chain in which the terminal methyl groups undergo a certain amount of thermal motion. One of the six-membered rings (C1-C5,C10) adopts a chair conformation, with puckering parameters (Cremer & Pople, 1975) of Q = 0.559 (4) Å, θ = 0.1 (4) ° and φ = 109 (19) °, the second six-membered ring (C5-C10) adopts a half-chair conformation, with puckering parameters Q = 0.500 (4) Å, θ = 51.1 (5) ° and φ = 326.3 (6) °, while the third six-membered ring (C8,C9,C11-C14) adopts a boat conformation, the puckering parameters are Q = 0.541 (4) Å, θ = 13.1 (4) ° and φ = 233 (2) °. The five-membered ring (C13-C17) has an envelope conformation on atom C13 (Baginski *et al.*, 1989; Gonzalez *et al.*, 2002), the corresponding puckering parameters are Q = 0.435 (4) Å and φ = 187.6 (6) °.

In the crystal the molecules are linked, via the water molecule of crystallization, by classical O—H···O hydrogen bonds to form a two-dimensional network with base vectors [100] and [010] (Fig. 2 and Table 1).

### Experimental

The isolated halotolerant fugal strain *Cladosporium cladosporioides* PXP-49, was isolated from roots of *Rhizophora stylosa* collected in the mangrove conservation area of Wenchang, Hainan, China. The working strain was cultured under static conditions at 298 K for 45 days in two hundred 1 *L* conical flasks containing the liquid medium (300 ml/flask) composed of maltose (20 g/*L*), mannitol (20 g/*L*), glucose (10 g/*L*), monosodium glutamate (10 g/*L*), KH_2_PO_4_ (5 g/*L*), MgSO_4_ (0.3 g/*L*), yeast extract (3 g/*L*), corn steep liquor (1 g/*L*), NaCl (100 g/*L*) after adjusting its pH to 10.0. The fermented whole broth (60 *L*) was filtered through cheese cloth to separate into supernatant and mycelia. The mycelia was extracted three times with acetone. The acetone solution was concentrated under reduced pressure to afford an aqueous solution. The aqueous solution was extracted three times with ethyl acetate to give an ethyl acetate solution which was concentrated under reduced pressure to give a crude extract (28 g).The crude extract was subjected to chromatography over silica gel column using a stepwise gradient elution of CHCl_3_-petroleum ether (0–100%) and then MeOH-CHCl_3_ (0–50%), to yield five fractions (Fr.1-Fr.5). The Fr.2 was further purified by another silica gel column using a step gradient elution of petroleum ether:acetone, followed by chromatographing on a Sephadex LH-20 eluting with CHCl_3_—MeOH (1:1) to afford three subfractions (Fr.2–6-1-Fr.2–6-3). The title compound (5.5 mg) was purified by extensive preparative HPLC using MeOH-H_2_O (9:1) from Fr.2–6-1. The single crystals were obtained by slow evaporation of CHCl_3_—MeOH (1:1) solution at 298 K.

### Refinement

All the H-atoms were positioned geometrically and allowed to ride on their parent atom: O-H_water_ = 0.85 Å, O-H_hydroxyl_ = 0.82 Å, C-H = 0.93, 096, 0.97 and 0.98 Å for CH(allyl), CH_3_, CH_2_, and CH(methine) H-atoms, respectively, with *U*_iso_(H) = k × *U*_eq_(O,C), where k = 1.5 for OH_hydroxyl_ and CH_3_ H-atoms, and k = 1.2 for all other H-atoms. The terminal methyl groups of the side chain undergo a certain amount of thermal motion. As mentioned above, the absolute configuration could not be determined crystallographically and the Friedel pairs were merged. The absolute configuration was assigned from the measured optical rotation and reference to the literature.

### Crystal data

**Table:**

C_28_H_44_O_4_·H_2_O	*F*(000) = 508
*M**_r_* = 462.65	*D*_x_ = 1.106 Mg m^−^^3^
Monoclinic, *P*2_1_	Mo *K*α radiation, λ = 0.71073 Å
Hall symbol: P 2yb	Cell parameters from 1224 reflections
*a* = 6.7605 (8) Å	θ = 2.9–18.6°
*b* = 7.2626 (11) Å	µ = 0.07 mm^−^^1^
*c* = 28.461 (2) Å	*T* = 298 K
β = 96.083 (1)°	Needle, colourless
*V* = 1389.5 (3) Å^3^	0.40 × 0.35 × 0.17 mm
*Z* = 2	

### Data collection

**Table:**

Bruker SMART CCD area-detector diffractometer	2641 independent reflections
Radiation source: fine-focus sealed tube	1557 reflections with *I* > 2σ(*I*)
graphite	*R*_int_ = 0.057
φ and ω scans	θ_max_ = 25.0°, θ_min_ = 2.9°
Absorption correction: multi-scan (*SADABS*; Sheldrick, 1996)	*h* = −7→8
*T*_min_ = 0.971, *T*_max_ = 0.988	*k* = −8→8
7040 measured reflections	*l* = −22→33

### Refinement

**Table:**

Refinement on *F*^2^	Primary atom site location: structure-invariant direct methods
Least-squares matrix: full	Secondary atom site location: difference Fourier map
*R*[*F*^2^ > 2σ(*F*^2^)] = 0.051	Hydrogen site location: inferred from neighbouring sites
*wR*(*F*^2^) = 0.108	H-atom parameters constrained
*S* = 1.02	*w* = 1/[σ^2^(*F*_o_^2^) + (0.036*P*)^2^] where *P* = (*F*_o_^2^ + 2*F*_c_^2^)/3
2641 reflections	(Δ/σ)_max_ = 0.001
304 parameters	Δρ_max_ = 0.14 e Å^−^^3^
1 restraint	Δρ_min_ = −0.15 e Å^−^^3^

### Special details

**Table:**

Geometry. All e.s.d.'s (except the e.s.d. in the dihedral angle between two l.s. planes) are estimated using the full covariance matrix. The cell e.s.d.'s are taken into account individually in the estimation of e.s.d.'s in distances, angles and torsion angles; correlations between e.s.d.'s in cell parameters are only used when they are defined by crystal symmetry. An approximate (isotropic) treatment of cell e.s.d.'s is used for estimating e.s.d.'s involving l.s. planes.
Refinement. Refinement of *F*^2^ against ALL reflections. The weighted *R*-factor *wR* and goodness of fit *S* are based on *F*^2^, conventional *R*-factors *R* are based on *F*, with *F* set to zero for negative *F*^2^. The threshold expression of *F*^2^ > σ(*F*^2^) is used only for calculating *R*-factors(gt) *etc*. and is not relevant to the choice of reflections for refinement. *R*-factors based on *F*^2^ are statistically about twice as large as those based on *F*, and *R*- factors based on ALL data will be even larger.

### Fractional atomic coordinates and isotropic or equivalent isotropic displacement parameters (Å^2^)

**Table:**

	*x*	*y*	*z*	*U*_iso_*/*U*_eq_	
O1	0.8875 (4)	0.0054 (4)	0.52563 (10)	0.0738 (10)	
H1	0.9909	0.0624	0.5235	0.111*	
O2	0.4476 (4)	0.2358 (4)	0.60388 (9)	0.0554 (8)	
H2	0.3863	0.2270	0.5775	0.083*	
O3	0.5562 (4)	0.6238 (4)	0.55751 (10)	0.0686 (9)	
O4	0.4840 (4)	0.2365 (4)	0.69723 (9)	0.0618 (8)	
H4	0.4212	0.2108	0.6718	0.093*	
O5	0.2328 (4)	0.1982 (4)	0.51822 (8)	0.0572 (8)	
H5C	0.1825	0.3000	0.5082	0.069*	
H5D	0.3038	0.1571	0.4976	0.069*	
C1	0.8286 (7)	0.1178 (6)	0.65380 (14)	0.0609 (12)	
H1A	0.9211	0.1058	0.6821	0.073*	
H1B	0.7149	0.0399	0.6576	0.073*	
C2	0.9293 (6)	0.0501 (6)	0.61159 (15)	0.0665 (13)	
H2A	1.0513	0.1188	0.6097	0.080*	
H2B	0.9640	−0.0787	0.6161	0.080*	
C3	0.7952 (6)	0.0726 (6)	0.56552 (15)	0.0595 (12)	
H3	0.6758	−0.0019	0.5680	0.071*	
C4	0.7289 (6)	0.2714 (6)	0.55897 (13)	0.0515 (11)	
H4A	0.6375	0.2816	0.5304	0.062*	
H4B	0.8437	0.3478	0.5551	0.062*	
C5	0.6271 (6)	0.3417 (5)	0.60092 (13)	0.0469 (11)	
C6	0.5590 (6)	0.5400 (6)	0.59528 (15)	0.0506 (11)	
C7	0.4882 (6)	0.6255 (6)	0.63688 (13)	0.0513 (11)	
H7	0.4196	0.7364	0.6331	0.062*	
C8	0.5173 (6)	0.5519 (5)	0.67999 (14)	0.0454 (10)	
C9	0.6322 (6)	0.3742 (5)	0.69000 (14)	0.0481 (11)	
C10	0.7588 (6)	0.3185 (5)	0.64904 (14)	0.0477 (11)	
C11	0.7618 (7)	0.3829 (6)	0.73789 (14)	0.0615 (13)	
H11A	0.8054	0.2592	0.7467	0.074*	
H11B	0.8795	0.4554	0.7340	0.074*	
C12	0.6586 (7)	0.4650 (6)	0.77811 (14)	0.0663 (13)	
H12A	0.7531	0.4736	0.8061	0.080*	
H12B	0.5519	0.3838	0.7852	0.080*	
C13	0.5735 (6)	0.6555 (6)	0.76590 (13)	0.0510 (11)	
C14	0.4259 (6)	0.6303 (6)	0.72149 (13)	0.0469 (10)	
H14	0.3312	0.5369	0.7300	0.056*	
C15	0.3080 (6)	0.8098 (6)	0.71665 (14)	0.0564 (12)	
H15A	0.1746	0.7887	0.7014	0.068*	
H15B	0.3738	0.8996	0.6984	0.068*	
C16	0.3026 (7)	0.8755 (6)	0.76823 (15)	0.0661 (13)	
H16A	0.1674	0.8727	0.7767	0.079*	
H16B	0.3523	1.0005	0.7719	0.079*	
C17	0.4362 (6)	0.7424 (6)	0.80017 (14)	0.0596 (12)	
H17	0.3503	0.6441	0.8101	0.072*	
C18	0.5296 (7)	0.8390 (7)	0.84525 (15)	0.0718 (14)	
H18	0.6067	0.9439	0.8354	0.086*	
C19	0.3696 (8)	0.9138 (8)	0.87252 (16)	0.0796 (15)	
H19	0.2887	0.8264	0.8848	0.096*	
C20	0.3289 (8)	1.0835 (8)	0.88128 (17)	0.0869 (16)	
H20	0.4126	1.1709	0.8700	0.104*	
C21	0.1657 (9)	1.1575 (9)	0.90707 (19)	0.1016 (19)	
H21	0.1019	1.0547	0.9219	0.122*	
C22	0.2406 (12)	1.2938 (10)	0.9449 (2)	0.131 (3)	
H22	0.3067	1.3949	0.9299	0.157*	
C23	0.0845 (14)	1.3730 (13)	0.9714 (3)	0.211 (4)	
H23A	0.0014	1.2760	0.9811	0.316*	
H23B	0.0052	1.4578	0.9516	0.316*	
H23C	0.1454	1.4366	0.9988	0.316*	
C24	0.9393 (6)	0.4470 (6)	0.64853 (14)	0.0608 (12)	
H24A	1.0417	0.4093	0.6725	0.091*	
H24B	0.9000	0.5711	0.6546	0.091*	
H24C	0.9888	0.4409	0.6182	0.091*	
C25	0.7358 (6)	0.7927 (6)	0.75607 (15)	0.0681 (14)	
H25A	0.7981	0.7527	0.7291	0.102*	
H25B	0.8335	0.7996	0.7831	0.102*	
H25C	0.6775	0.9120	0.7499	0.102*	
C26	0.6712 (9)	0.7170 (9)	0.87720 (17)	0.110 (2)	
H26A	0.7094	0.7798	0.9064	0.165*	
H26B	0.7876	0.6909	0.8617	0.165*	
H26C	0.6055	0.6038	0.8835	0.165*	
C27	0.0112 (11)	1.2509 (12)	0.8717 (2)	0.186 (4)	
H27A	0.0681	1.3595	0.8593	0.280*	
H27B	−0.1028	1.2843	0.8872	0.280*	
H27C	−0.0285	1.1675	0.8462	0.280*	
C28	0.3933 (12)	1.1993 (12)	0.9799 (2)	0.175 (3)	
H28A	0.3376	1.0880	0.9910	0.262*	
H28B	0.4293	1.2800	1.0061	0.262*	
H28C	0.5095	1.1705	0.9646	0.262*	

### Atomic displacement parameters (Å^2^)

**Table:**

	*U*^11^	*U*^22^	*U*^33^	*U*^12^	*U*^13^	*U*^23^
O1	0.081 (2)	0.070 (2)	0.076 (2)	−0.0171 (19)	0.0347 (17)	−0.0278 (17)
O2	0.0557 (18)	0.0629 (19)	0.0479 (16)	−0.0209 (16)	0.0069 (13)	−0.0030 (14)
O3	0.092 (2)	0.068 (2)	0.0496 (18)	0.0010 (18)	0.0244 (17)	0.0116 (16)
O4	0.081 (2)	0.0486 (18)	0.0589 (18)	−0.0105 (17)	0.0215 (16)	0.0065 (14)
O5	0.0665 (19)	0.0589 (18)	0.0477 (16)	0.0020 (15)	0.0129 (14)	0.0026 (14)
C1	0.073 (3)	0.053 (3)	0.058 (3)	0.005 (3)	0.010 (2)	−0.002 (2)
C2	0.073 (3)	0.055 (3)	0.075 (3)	0.007 (3)	0.022 (3)	−0.002 (3)
C3	0.062 (3)	0.060 (3)	0.061 (3)	−0.005 (2)	0.023 (2)	−0.014 (2)
C4	0.053 (3)	0.056 (3)	0.048 (3)	−0.013 (2)	0.012 (2)	−0.007 (2)
C5	0.054 (3)	0.041 (3)	0.047 (3)	−0.011 (2)	0.011 (2)	−0.001 (2)
C6	0.056 (3)	0.047 (3)	0.049 (3)	−0.011 (2)	0.009 (2)	0.004 (2)
C7	0.062 (3)	0.048 (3)	0.046 (3)	0.000 (2)	0.013 (2)	0.001 (2)
C8	0.050 (3)	0.041 (3)	0.045 (3)	−0.010 (2)	0.006 (2)	−0.001 (2)
C9	0.055 (3)	0.042 (3)	0.048 (3)	0.001 (2)	0.009 (2)	0.005 (2)
C10	0.056 (3)	0.041 (2)	0.047 (3)	−0.004 (2)	0.010 (2)	−0.0045 (19)
C11	0.075 (3)	0.060 (3)	0.049 (3)	0.012 (3)	0.002 (2)	−0.003 (2)
C12	0.084 (3)	0.066 (3)	0.048 (3)	0.008 (3)	0.004 (2)	0.000 (2)
C13	0.058 (3)	0.054 (3)	0.042 (2)	−0.006 (2)	0.008 (2)	−0.004 (2)
C14	0.049 (2)	0.046 (3)	0.047 (2)	−0.009 (2)	0.009 (2)	−0.001 (2)
C15	0.060 (3)	0.054 (3)	0.056 (3)	−0.003 (2)	0.009 (2)	−0.007 (2)
C16	0.065 (3)	0.071 (3)	0.065 (3)	−0.008 (3)	0.016 (3)	−0.017 (3)
C17	0.066 (3)	0.061 (3)	0.054 (3)	−0.003 (3)	0.014 (2)	−0.007 (2)
C18	0.087 (4)	0.078 (3)	0.051 (3)	0.003 (3)	0.008 (3)	−0.012 (3)
C19	0.101 (4)	0.084 (4)	0.056 (3)	0.005 (4)	0.018 (3)	−0.014 (3)
C20	0.111 (4)	0.089 (4)	0.063 (3)	0.007 (4)	0.016 (3)	−0.018 (3)
C21	0.125 (5)	0.109 (5)	0.072 (4)	0.042 (4)	0.017 (4)	−0.019 (4)
C22	0.160 (7)	0.132 (6)	0.102 (5)	0.053 (5)	0.025 (5)	−0.033 (5)
C23	0.236 (10)	0.210 (10)	0.191 (9)	0.069 (9)	0.052 (7)	−0.078 (8)
C24	0.058 (3)	0.065 (3)	0.060 (3)	−0.008 (3)	0.007 (2)	−0.013 (2)
C25	0.059 (3)	0.082 (4)	0.064 (3)	−0.009 (3)	0.009 (2)	−0.020 (3)
C26	0.128 (5)	0.132 (5)	0.066 (3)	0.040 (5)	−0.008 (3)	−0.022 (4)
C27	0.166 (7)	0.252 (11)	0.135 (6)	0.113 (8)	−0.015 (5)	−0.017 (7)
C28	0.214 (9)	0.195 (9)	0.111 (5)	0.036 (8)	−0.004 (6)	−0.038 (6)

### Geometric parameters (Å, °)

**Table:**

O1—C3	1.438 (4)	C14—H14	0.9800
O1—H1	0.8200	C15—C16	1.548 (5)
O2—C5	1.447 (4)	C15—H15A	0.9700
O2—H2	0.8200	C15—H15B	0.9700
O3—C6	1.234 (4)	C16—C17	1.550 (6)
O4—C9	1.446 (5)	C16—H16A	0.9700
O4—H4	0.8200	C16—H16B	0.9700
O5—H5C	0.8500	C17—C18	1.538 (5)
O5—H5D	0.8500	C17—H17	0.9800
C1—C2	1.524 (5)	C18—C19	1.498 (6)
C1—C10	1.533 (5)	C18—C26	1.531 (7)
C1—H1A	0.9700	C18—H18	0.9800
C1—H1B	0.9700	C19—C20	1.293 (6)
C2—C3	1.522 (6)	C19—H19	0.9300
C2—H2A	0.9700	C20—C21	1.489 (7)
C2—H2B	0.9700	C20—H20	0.9300
C3—C4	1.517 (6)	C21—C22	1.510 (8)
C3—H3	0.9800	C21—C27	1.531 (8)
C4—C5	1.528 (5)	C21—H21	0.9800
C4—H4A	0.9700	C22—C23	1.478 (9)
C4—H4B	0.9700	C22—C28	1.520 (9)
C5—C6	1.516 (6)	C22—H22	0.9800
C5—C10	1.561 (5)	C23—H23A	0.9600
C6—C7	1.461 (5)	C23—H23B	0.9600
C7—C8	1.334 (5)	C23—H23C	0.9600
C7—H7	0.9300	C24—H24A	0.9600
C8—C14	1.502 (5)	C24—H24B	0.9600
C8—C9	1.518 (5)	C24—H24C	0.9600
C9—C11	1.542 (5)	C25—H25A	0.9600
C9—C10	1.571 (5)	C25—H25B	0.9600
C10—C24	1.537 (5)	C25—H25C	0.9600
C11—C12	1.524 (5)	C26—H26A	0.9600
C11—H11A	0.9700	C26—H26B	0.9600
C11—H11B	0.9700	C26—H26C	0.9600
C12—C13	1.524 (6)	C27—H27A	0.9600
C12—H12A	0.9700	C27—H27B	0.9600
C12—H12B	0.9700	C27—H27C	0.9600
C13—C25	1.529 (6)	C28—H28A	0.9600
C13—C14	1.536 (5)	C28—H28B	0.9600
C13—C17	1.551 (5)	C28—H28C	0.9600
C14—C15	1.527 (5)		
			
C3—O1—H1	109.5	C14—C15—C16	104.0 (3)
C5—O2—H2	109.5	C14—C15—H15A	111.0
C9—O4—H4	109.5	C16—C15—H15A	111.0
H5C—O5—H5D	108.0	C14—C15—H15B	111.0
C2—C1—C10	113.3 (4)	C16—C15—H15B	111.0
C2—C1—H1A	108.9	H15A—C15—H15B	109.0
C10—C1—H1A	108.9	C15—C16—C17	107.1 (3)
C2—C1—H1B	108.9	C15—C16—H16A	110.3
C10—C1—H1B	108.9	C17—C16—H16A	110.3
H1A—C1—H1B	107.7	C15—C16—H16B	110.3
C3—C2—C1	111.7 (4)	C17—C16—H16B	110.3
C3—C2—H2A	109.3	H16A—C16—H16B	108.6
C1—C2—H2A	109.3	C18—C17—C16	111.5 (4)
C3—C2—H2B	109.3	C18—C17—C13	119.3 (4)
C1—C2—H2B	109.3	C16—C17—C13	103.6 (3)
H2A—C2—H2B	107.9	C18—C17—H17	107.3
O1—C3—C4	111.9 (4)	C16—C17—H17	107.3
O1—C3—C2	112.0 (3)	C13—C17—H17	107.3
C4—C3—C2	110.4 (3)	C19—C18—C26	110.0 (4)
O1—C3—H3	107.4	C19—C18—C17	110.0 (4)
C4—C3—H3	107.4	C26—C18—C17	114.0 (4)
C2—C3—H3	107.4	C19—C18—H18	107.5
C3—C4—C5	111.9 (3)	C26—C18—H18	107.5
C3—C4—H4A	109.2	C17—C18—H18	107.5
C5—C4—H4A	109.2	C20—C19—C18	128.7 (6)
C3—C4—H4B	109.2	C20—C19—H19	115.6
C5—C4—H4B	109.2	C18—C19—H19	115.6
H4A—C4—H4B	107.9	C19—C20—C21	128.5 (6)
O2—C5—C6	105.3 (3)	C19—C20—H20	115.7
O2—C5—C4	108.5 (3)	C21—C20—H20	115.7
C6—C5—C4	113.1 (3)	C20—C21—C22	112.2 (5)
O2—C5—C10	107.2 (3)	C20—C21—C27	109.0 (5)
C6—C5—C10	109.5 (3)	C22—C21—C27	109.4 (6)
C4—C5—C10	112.8 (3)	C20—C21—H21	108.7
O3—C6—C7	121.4 (4)	C22—C21—H21	108.7
O3—C6—C5	122.5 (4)	C27—C21—H21	108.7
C7—C6—C5	116.1 (4)	C23—C22—C21	114.6 (7)
C8—C7—C6	123.1 (4)	C23—C22—C28	108.2 (7)
C8—C7—H7	118.4	C21—C22—C28	108.9 (6)
C6—C7—H7	118.4	C23—C22—H22	108.3
C7—C8—C14	122.6 (4)	C21—C22—H22	108.3
C7—C8—C9	122.4 (4)	C28—C22—H22	108.3
C14—C8—C9	114.8 (3)	C22—C23—H23A	109.5
O4—C9—C8	105.4 (3)	C22—C23—H23B	109.5
O4—C9—C11	103.9 (3)	H23A—C23—H23B	109.5
C8—C9—C11	111.3 (3)	C22—C23—H23C	109.5
O4—C9—C10	111.6 (3)	H23A—C23—H23C	109.5
C8—C9—C10	112.8 (3)	H23B—C23—H23C	109.5
C11—C9—C10	111.3 (3)	C10—C24—H24A	109.5
C1—C10—C24	109.9 (3)	C10—C24—H24B	109.5
C1—C10—C5	108.8 (3)	H24A—C24—H24B	109.5
C24—C10—C5	107.8 (3)	C10—C24—H24C	109.5
C1—C10—C9	111.5 (3)	H24A—C24—H24C	109.5
C24—C10—C9	110.2 (3)	H24B—C24—H24C	109.5
C5—C10—C9	108.6 (3)	C13—C25—H25A	109.5
C12—C11—C9	114.7 (4)	C13—C25—H25B	109.5
C12—C11—H11A	108.6	H25A—C25—H25B	109.5
C9—C11—H11A	108.6	C13—C25—H25C	109.5
C12—C11—H11B	108.6	H25A—C25—H25C	109.5
C9—C11—H11B	108.6	H25B—C25—H25C	109.5
H11A—C11—H11B	107.6	C18—C26—H26A	109.5
C11—C12—C13	112.1 (3)	C18—C26—H26B	109.5
C11—C12—H12A	109.2	H26A—C26—H26B	109.5
C13—C12—H12A	109.2	C18—C26—H26C	109.5
C11—C12—H12B	109.2	H26A—C26—H26C	109.5
C13—C12—H12B	109.2	H26B—C26—H26C	109.5
H12A—C12—H12B	107.9	C21—C27—H27A	109.5
C12—C13—C25	111.9 (4)	C21—C27—H27B	109.5
C12—C13—C14	106.0 (3)	H27A—C27—H27B	109.5
C25—C13—C14	110.1 (3)	C21—C27—H27C	109.5
C12—C13—C17	117.5 (3)	H27A—C27—H27C	109.5
C25—C13—C17	109.8 (3)	H27B—C27—H27C	109.5
C14—C13—C17	100.7 (3)	C22—C28—H28A	109.5
C8—C14—C15	120.5 (3)	C22—C28—H28B	109.5
C8—C14—C13	114.0 (3)	H28A—C28—H28B	109.5
C15—C14—C13	105.1 (3)	C22—C28—H28C	109.5
C8—C14—H14	105.3	H28A—C28—H28C	109.5
C15—C14—H14	105.3	H28B—C28—H28C	109.5
C13—C14—H14	105.3		
			
C10—C1—C2—C3	−56.2 (5)	C8—C9—C10—C5	−45.5 (4)
C1—C2—C3—O1	−178.8 (3)	C11—C9—C10—C5	−171.4 (3)
C1—C2—C3—C4	55.8 (5)	O4—C9—C11—C12	−69.0 (4)
O1—C3—C4—C5	178.9 (3)	C8—C9—C11—C12	44.0 (5)
C2—C3—C4—C5	−55.6 (4)	C10—C9—C11—C12	170.8 (4)
C3—C4—C5—O2	−63.5 (4)	C9—C11—C12—C13	−54.7 (5)
C3—C4—C5—C6	−179.9 (3)	C11—C12—C13—C25	−61.0 (5)
C3—C4—C5—C10	55.2 (4)	C11—C12—C13—C14	59.0 (5)
O2—C5—C6—O3	−106.8 (4)	C11—C12—C13—C17	170.6 (4)
C4—C5—C6—O3	11.5 (5)	C7—C8—C14—C15	−5.1 (6)
C10—C5—C6—O3	138.2 (4)	C9—C8—C14—C15	179.3 (3)
O2—C5—C6—C7	70.7 (4)	C7—C8—C14—C13	−131.5 (4)
C4—C5—C6—C7	−170.9 (3)	C9—C8—C14—C13	53.0 (4)
C10—C5—C6—C7	−44.3 (5)	C12—C13—C14—C8	−58.9 (4)
O3—C6—C7—C8	−169.0 (4)	C25—C13—C14—C8	62.3 (5)
C5—C6—C7—C8	13.4 (6)	C17—C13—C14—C8	178.2 (3)
C6—C7—C8—C14	−173.5 (3)	C12—C13—C14—C15	167.1 (3)
C6—C7—C8—C9	1.7 (6)	C25—C13—C14—C15	−71.7 (4)
C7—C8—C9—O4	−106.3 (4)	C17—C13—C14—C15	44.1 (4)
C14—C8—C9—O4	69.2 (4)	C8—C14—C15—C16	−161.5 (3)
C7—C8—C9—C11	141.6 (4)	C13—C14—C15—C16	−31.2 (4)
C14—C8—C9—C11	−42.8 (4)	C14—C15—C16—C17	5.9 (4)
C7—C8—C9—C10	15.7 (5)	C15—C16—C17—C18	150.7 (4)
C14—C8—C9—C10	−168.7 (3)	C15—C16—C17—C13	21.1 (4)
C2—C1—C10—C24	−64.8 (4)	C12—C13—C17—C18	81.5 (5)
C2—C1—C10—C5	53.0 (5)	C25—C13—C17—C18	−47.9 (5)
C2—C1—C10—C9	172.7 (3)	C14—C13—C17—C18	−164.0 (4)
O2—C5—C10—C1	67.0 (4)	C12—C13—C17—C16	−153.8 (4)
C6—C5—C10—C1	−179.2 (3)	C25—C13—C17—C16	76.8 (4)
C4—C5—C10—C1	−52.4 (4)	C14—C13—C17—C16	−39.3 (4)
O2—C5—C10—C24	−173.8 (3)	C16—C17—C18—C19	58.6 (5)
C6—C5—C10—C24	−60.1 (4)	C13—C17—C18—C19	179.4 (4)
C4—C5—C10—C24	66.8 (4)	C16—C17—C18—C26	−177.3 (4)
O2—C5—C10—C9	−54.4 (4)	C13—C17—C18—C26	−56.5 (6)
C6—C5—C10—C9	59.3 (4)	C26—C18—C19—C20	119.0 (6)
C4—C5—C10—C9	−173.8 (3)	C17—C18—C19—C20	−114.6 (6)
O4—C9—C10—C1	−46.7 (4)	C18—C19—C20—C21	177.8 (5)
C8—C9—C10—C1	−165.2 (3)	C19—C20—C21—C22	130.0 (7)
C11—C9—C10—C1	68.8 (4)	C19—C20—C21—C27	−108.7 (8)
O4—C9—C10—C24	−169.1 (3)	C20—C21—C22—C23	179.5 (6)
C8—C9—C10—C24	72.4 (4)	C27—C21—C22—C23	58.5 (9)
C11—C9—C10—C24	−53.5 (4)	C20—C21—C22—C28	−59.1 (8)
O4—C9—C10—C5	73.0 (4)	C27—C21—C22—C28	179.8 (6)

### Hydrogen-bond geometry (Å, °)

**Table:**

*D*—H···*A*	*D*—H	H···*A*	*D*···*A*	*D*—H···*A*
O1—H1···O5^i^	0.82	1.93	2.749 (4)	180
O2—H2···O5	0.82	1.90	2.716 (4)	177
O4—H4···O2	0.82	1.97	2.642 (4)	139
O5—H5C···O1^ii^	0.85	1.81	2.642 (4)	165
O5—H5D···O3^iii^	0.85	1.93	2.761 (4)	165

Symmetry codes: (i) *x*+1, *y*, *z*; (ii) −*x*+1, *y*+1/2, −*z*+1; (iii) −*x*+1, *y*−1/2, −*z*+1.
